# Crude Polysaccharide Extracted From *Moringa oleifera* Leaves Prevents Obesity in Association With Modulating Gut Microbiota in High-Fat Diet-Fed Mice

**DOI:** 10.3389/fnut.2022.861588

**Published:** 2022-04-25

**Authors:** Lingfei Li, Li Ma, Yanlong Wen, Jing Xie, Liang Yan, Aibing Ji, Yin Zeng, Yang Tian, Jun Sheng

**Affiliations:** ^1^College of Food Science and Technology, Yunnan Agricultural University, Kunming, China; ^2^Engineering Research Center of Development and Utilization of Food and Drug Homologous Resources, Ministry of Education, Yunnan Agricultural University, Kunming, China; ^3^Pu'er Institute of Pu-erh Tea, Pu'er, China; ^4^College of Tea (Pu'er), West Yunnan University of Applied Sciences, Pu'er, China; ^5^Key Laboratory of Pu-er Tea Science, Ministry of Education, Yunnan Agricultural University, Kunming, China

**Keywords:** polysaccharide, *Moringa oleifera* leaves, gut microbiota, obesity, high-fat diet

## Abstract

*Moringa oleifera* is a commonly used plant with high nutritional and medicinal values. *M. oleifera* leaves are considered a new food resource in China. However, the biological activities of *M. oleifera* polysaccharides (MOP) in regulating gut microbiota and alleviating obesity remain obscure. In the present study, we prepared the MOP and evaluated its effects on obesity and gut microbiota in high-fat diet (HFD)-induced C57BL/6J mice. The experimental mice were supplemented with a normal chow diet (NCD group), a high-fat diet (HFD group), and HFD along with MOP at a different dose of 100, 200, and 400 mg/kg/d, respectively. Physiological, histological, biochemical parameters, genes related to lipid metabolism, and gut microbiota composition were compared among five experimental groups. The results showed that MOP supplementation effectively prevented weight gain and lipid accumulation induced by HFD, ameliorated blood lipid levels and insulin resistance, alleviated the secretion of pro-inflammatory cytokines, and regulated the expression of genes related to lipid metabolism and bile acid metabolism. In addition, MOP positively reshaped the gut microbiota composition, significantly increasing the abundance of *Bacteroides*, norank_f_Ruminococcaceae, and *Oscillibacter*, while decreasing the relative abundance of *Blautia, Alistipes*, and *Tyzzerella*, which are closely associated with obesity. These results demonstrated that MOP supplementation has a protective effect against HFD-induced obesity in mice, which was associated with reshaping the gut microbiota. To the best of our knowledge, this is the first report on the potential of MOP to prevent obesity and modulating gut microbiota, which suggests that MOP can be used as a potential prebiotic.

## Introduction

In recent years, the prevalence of obesity has increased dramatically and has become a severe health problem worldwide. In 2016, 39% (more than 1.9 billion) of adults were overweight, and 13% were obese (1). The worldwide prevalence of obesity nearly tripled between 1975 and 2016 (2). It has been estimated that by 2030, the obesity rate will reach up to 20% of the adult population. Approximately 500 million people are obese, and 1.4 billion are overweight globally (3). The main characteristics of obesity are excessive weight gain and fat accumulation due to various factors, such as genetic predisposition, high-calorie energy intake, and sedentary lifestyle (4). Obesity can cause chronic low-grade inflammation, hyperglycemia, hyperlipidemia, and insulin resistance (5, 6). Accumulating evidence suggests that obesity is associated with various metabolic disorders, such as cardiovascular disease, type II diabetes, non-alcoholic fatty liver disease, and various kinds of cancers (3). With the recognition that obesity is responsible for a growing prevalence of chronic diseases, obesity has been recognized as “the greatest threat to global public health in this century” (7). Reducing and curbing obesity is one of the most important health challenges in the modern world. Depending on the severity of the disease, there are several ways to intervene or treat obesity, including lifestyle changes, pharmacotherapy, and bariatric surgery (8). However, the use of drugs or bariatric surgery has been shown to be associated with significant side effects, some of which could increase the risk of developing chronic diseases (6, 9). Therefore, the search for natural compounds to treat obesity and its related diseases has attracted much research interest.

Despite significant research efforts to combat obesity-induced metabolic syndrome in the last decade, there has been slow progress in understanding the causes and mechanisms that regulate its development. In recent years, accumulating evidence suggests that gut microbiota dysbiosis is strongly associated with host metabolism and obesity development (10–12). Studies have shown that the high-fat diet can change the structure of gut microbiota, disrupt the intestinal microenvironment, and lead to dysbiosis of gut microbiota (13, 14). Conversely, it has been shown that gut microbiota play an important role in the dynamic balance of metabolism by regulating intestinal endothelial barrier function, glucose metabolism, and chronic inflammation associated with obesity. Therefore, it has been proposed that altering the composition of gut microbiota by dietary or other means can confer beneficial effects, restoring the integrity of intestinal function and reversing the characteristics of obesity (15).

In recent years, polysaccharides have attracted attention for their role in weight loss. Polysaccharides are macromolecular polymers composed of at least ten monosaccharides linked by glycosidic bonds (16), and they are natural macromolecular active substances widely found in animals, plants, algae, and microbial cells (17). Polysaccharides are well-known for their health benefits, such as immunomodulatory (18), anti-tumor (19), anti-inflammatory (20), anti-oxidant (21), and hypolipidemic effects (22). Previous studies have suggested that polysaccharides from plants, such as *Angelica sinensis* (23), *Schisandra* (24), and *Ophiopogon* (25), show great lipid-lowering effects. Polysaccharides can reduce body weight, leaky gut, and low-grade inflammation in various tissues by physicochemical properties, such as water retention, and/or by probiotic activity, such as altering gut microbiota and production of microbiota-derived metabolites (26). A study showed that *Polygonatum odoratum* polysaccharides modulated gut microbiota and mitigated obesity induced by a high-fat diet (HFD) in rats (27). It has also been shown that the phylum Firmicutes was 40% higher than the phylum Bacteroidetes in HFD-fed mice, and supplementation with *Ophiopogon* polysaccharides increased Bacteroidetes by 28% and decreased Firmicutes by 15% (25). Thus, dietary polysaccharides are considered one of the effective regulators of gut microbiota, which may help the host regulate metabolism.

*Moringa oleifera* Lam. is a perennial plant of the Moringaceae family, also known as the “drumstick tree,” “miracle tree,” or “tree of life” (28). *M. oleifera* is rich in nutrients, including protein, vitamins, essential amino acids, minerals, dietary fiber, and bioactive compounds (29). In 2011, the Ministry of Health of the People's Republic of China issued a notice to consider *M. oleifera l*eaves as a new food resource. It has been demonstrated that *M. oleifera* has many biological functions such as anti-microbial, anti-oxidant, anti-inflammatory, anti-cancer, hepatoprotective, hypoglycemic, hypolipidemic, and other effects (30–32). However, the biological activities of *M. oleifera* polysaccharides (MOP) in regulating gut microbiota and alleviating obesity have not yet been reported. We hypothesized that MOP could positively modulate the gut microbiota and attenuate HFD-induced obesity. Therefore, to verify this hypothesis, we investigated the effects of MOP on body weight, fat accumulation, blood lipid levels, insulin resistance, chronic inflammation, and gut microbiota in a HFD-induced obese mice, and further investigated the correlation of gut bacteria with obesity-related parameters including host obesity phenotype, blood lipids, glucose homeostasis, and pro-inflammatory cytokines.

## Materials and Methods

### Preparation of Crude Polysaccharides From *M. oleifera* Leaves (MOP)

*M. oleifera* leaf powder was purchased from Yunnan Tianyou Technology Development Co. (Dehong, China). The crude polysaccharide was extracted according to the methods described in previous studies (33). In brief, *M. oleifera* leaf powder was extracted three times with deionized water at a ratio of 1:10 (w/v) at 70°C for 90 min, followed by centrifuging at 4,000 rpm for 20 min. The supernatants were combined and concentrated by rotary evaporation. The concentrates were added to anhydrous ethanol to obtain a final ethanol concentration of 80% (v/v), and kept at 4°C overnight. The resulting precipitates were gained after centrifugation, washed with 95% ethanol, dissolved in deionized water, then loaded into dialysis bags (molecular weight cutoff: 3,500 Da) and dialyzed for 2 days at 4°C, and the deionized water was changed every 4 h. The dialyzed solution was vacuum freeze-dried to obtain the crude polysaccharide of *M. oleifera* (MOP). The polysaccharide content of MOP was measured by the phenol-sulfuric acid method (34). Total protein content was quantified by the Bradford method (35). Dietary fiber concentrations were determined according to the method of Prosky et al. (36). The monosaccharide composition was analyzed by the high-performance liquid chromatography (HPLC) technique (37). Chemical and monosaccharide compositions of MOP are shown in Table 1 and Supplementary Figure 1.

**Table 1:** Chemical and monosaccharides compositions of MOP.

**Chemical composition**	**Value**
Total sugar (%)	38.90
Total dietary fiber (g/100 g)	48.70
Protein (g/100 g)	3.88
Mannose (g/kg)	10.81
Ribose (g/kg)	1.01
Rhamnose (g/kg)	22.42
Glucuronic acid (g/kg)	2.14
Galacturonic acid (g/kg)	34.25
Glucose (g/kg)	47.60
Galactose (g/kg)	252.72
Xylose (g/kg)	7.29
Arabinose (g/kg)	111.16
Fucose (g/kg)	2.53

### Animals and Diets

Seven-week-old male C57BL/6J mice were purchased from Kunming Medical University Laboratory Animal Center (Kunming, China). All mice were maintained in controlled environmental conditions (a 12/12 h light/dark cycle, 25 ± 1°C, and 55% humidity) with free access to food and water. After acclimation for 1 week, the mice were randomly divided into five groups (*n* = 8, each group) to be fed the following diets for 12 weeks: (1) NCD (normal chow diet, TP23302, 10% calories from fat, Trophic Animal Feed High-tech Co., Ltd, Nantong, China), which was daily gavaged with distilled water only, as control; (2) HFD (high-fat diet, TP23300, 60% calories from fat, Trophic Animal Feed High-tech Co., Ltd, Nantong, China), which was daily gavaged with distilled water only, as control; (3) HFD + MOP100 (gavaged with 100 mg/kg/d MOP); (4) HFD + MOP200 (gavaged with 200 mg/kg/d MOP); (5) HFD + MOP400 (gavaged with 400 mg/kg/d MOP). The animal protocol used in this study was approved by the Animal Care and Use Committee of Yunnan Agricultural University.

### Blood and Tissue Sample Collection

At the end of the experiment, the mice were anesthetized with 3% pentobarbital sodium after 12 h of fasting. The blood samples were collected from the retro-orbital sinus and centrifuged at 12,000 rpm for 10 min at 4°C to obtain serum. Perirenal fat, mesenteric fat, and liver from each mouse were collected and weighted. A portion of the liver and perirenal adipose tissues were immersion-fixed in 10% neutral formalin. The remaining tissues and cecum contents were snap-frozen in liquid nitrogen within 10 min postmortem, then preserved at −80°C.

### Histopathological Examination

Formalin-fixed liver and perirenal adipose tissues were embedded in paraffin, cut into 5-μm sections, stained with hematoxylin and eosin (H & E), and visualized under a microscope (Olympus CX43, Japan). Adipocyte size was measured using ImageJ software.

### Biochemical Analysis

Serum total cholesterol (T-CHO), triacylglycerol (TG), low-density lipoprotein cholesterol (LDL-C), and high-density lipoprotein cholesterol (HDL-C) were measured with commercial assay kits (Nanjing Jiancheng Bioengineering Institute, China), respectively. Fasting blood glucose was determined by the glucose oxidase method using the Glucose GOD-PAD kit (Rongsheng Biotech Co. Ltd, Shanghai, China). Serum insulin levels were measured using the mouse insulin ELISA kit (Beijing Solarbio Science & Technology Co., China). Serum TNF-α and IL-1β were measured using ELISA kits (Beijing 4A Biotech Co., China). All protocols were performed according to the manufacturers' instructions. The homeostasis model assessment of insulin resistance (HOMA-IR) index was calculated using the formula (HOMA-IR = fasting insulin (mU/l) × fasting glucose (mmol/l)/22.5 (38).

### Quantitative PCR Analysis of Gene Expression

Total RNA was extracted from liver tissue using Trizol reagent (Takara, Dalian, China), and quantified using a NanoDrop 2000 Spectrophotometer (Thermo Fisher, USA). Complementary DNA was synthesized using the PrimeScript^TM^ RT reagent kit with a genomic DNA Eraser (Takara, Dalian, China) in accordance with the manufacturer's protocol. Real-time quantitative polymerase chain reaction (RT-qPCR) was performed with SYBR Premix Ex Taq^TM^II (Takara, Dalian, China) in the Bio-Rad CFX96 Thermocycler (Bio-Rad, USA). All the primers are listed in Supplementary Table 1.

### Gut Microbiota Analysis

Bacterial genomic DNA was extracted from cecal contents using the QIAamp-DNA Stool Mini Kit (Qiagen, Hilden, Germany) according to the manufacturer's instructions. DNA samples were sent to Majorbio Biotechnology Co., Ltd. (Shanghai, China) under dry ice conditions for 16S rRNA gene sequencing. The V3–V4 hypervariable regions of the 16S rRNA gene were amplified with primers 338F (5′-ACTCCTACGGGAGGCAGCAG-3′) and 806R (5′-GGACTACHVGGGTWTCTAAT-3′) using a thermocycler PCR system (GeneAmp 9700; ABI, USA). The raw reads were deposited into the NCBI Sequence Read Archive database (Accession: PRJNA759102).

Subsequent bioinformatics analysis was performed through the free online Majorbio I-Sanger Cloud Platform (www.i-sanger.com). Operational taxonomic units (OTUs) were picked with a 97% similarity threshold. In the analysis, alpha-diversity metrics were used, including Sobs (the actually observed richness), Chao (Chao index of species richness), ACE (the ACE index of species richness), and Shannon diversity index. Beta-diversity of gut microbiota, including hierarchical clustering tree and Principal Coordinate Analysis (PCoA), was performed based on the unweighted UniFrac distance. Moreover, the analysis of similarity (ANOSIM) test was used to test the significant differences between sample groupings. The linear discriminant analysis (LDA) effect size (LEfSe) was used to identify which bacterial taxa drove changes in the microbiota community, and the LDA threshold was >3.0). The two-factor correlation network analysis was performed to determine the correlations between specific gut bacteria and obesity-related biomarkers.

### Statistical Analysis

Statistical analysis was performed with SPSS statistics 19.0. Data sets involving more than two groups were analyzed using one-way ANOVA, followed by Duncan's multiple range tests. A value of *p* < 0.05 was considered to be statistically significant.

## Results

### MOP Attenuates Body Weight Gain Induced by HFD

**Figure 1:**
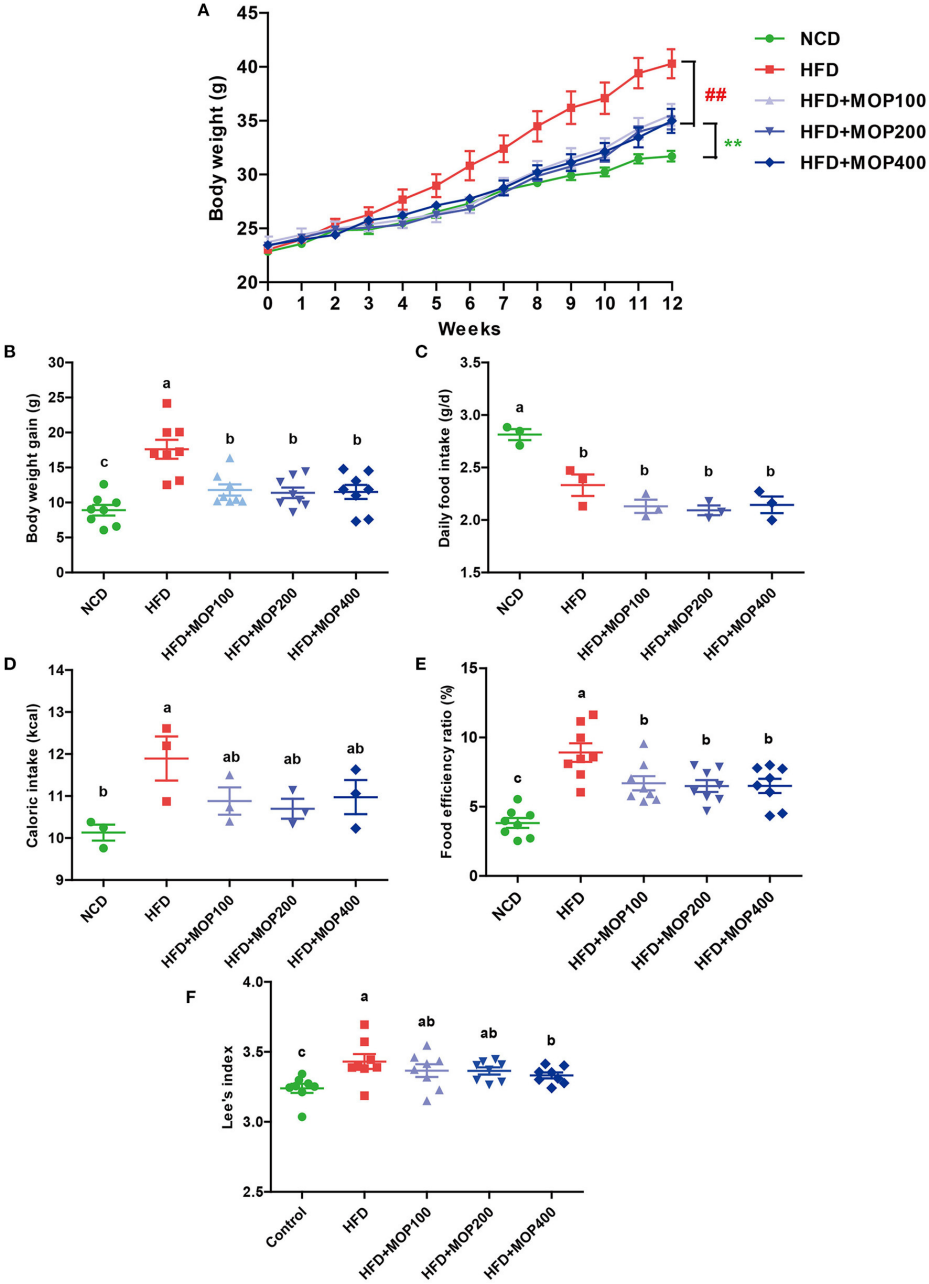
MOP reduces body weight in HFD-fed mice. **(A)** The change in body weight of the mice during the experiment (*n* = 8 mice/group); **(B)** body weight gain (*n* = 8); **(C)** daily food intake (*n* = 3 cages/group); **(D)** caloric intake (*n* = 3); **(E)** food efficiency ratio (EFR), food efficiency ratio (%) = total body weight gain/total food intake × 100 (*n* = 8); **(F)** Lee's index, Lee's index = body weight (g)∧(1/3) × 1,000/body length (cm) (*n* = 8). Data are shown as means ± SEM. Bars marked with different superscript letters (a–c) indicate significant differences at *p* < 0.05 based on one-way ANOVA with Duncan's *post-hoc* test.

As shown in Figure 1A, the mean initial body weight of the mice in the 5 groups ranged from 22.85 to 23.73 g. One-way ANOVA showed no statistical difference in mouse body weight between all diet groups at the beginning of the experiment (*F* = 0.8950, *p* = 0.4749). As expected, the mice fed with HFD gained significantly more weight than the NCD mice after 12 weeks (*p* < 0.01) (Figures 1A,B). Supplementation with MOP effectively attenuated the body weight gain in HFD-fed mice, although there was no significant difference between the different MOP dose groups (100, 200, and 400 mg/kg/d). Daily food intake and caloric intake were measured to determine whether MOP functions by modulating food or energy intake in mice. The data showed that the daily food intake and caloric intake of MOP-treated groups were not statistically different from those of the HFD group (Figures 1C,D), but their body weight gain was lower than that of mice fed HFD (*p* < 0.05) (Figure 1B), suggesting that the protective effect of MOP against HFD-induced weight gain was not through suppression of food or energy intake. Additionally, the food efficiency ratio (FER) in the HFD group was significantly higher than in the NCD group (*p* < 0.01), while supplementation with MOP significantly decreased the FER (Figure 1E). Lee's index can comprehensively reflect the proportional relationship between body weight and body length, which can be used as an indicator to evaluate the degree of obesity in obese model mice. In the present study, HFD significantly increased the Lee's index in mice, while MOP supplementation decreased the Lee's index (Figure 1F). These results suggest that MOP intervention is effective in attenuating body weight gain induced by HFD in mice.

### MOP Inhibits Adipose Hypertrophy and Liver Steatosis Induced by HFD

**Figure 2:**
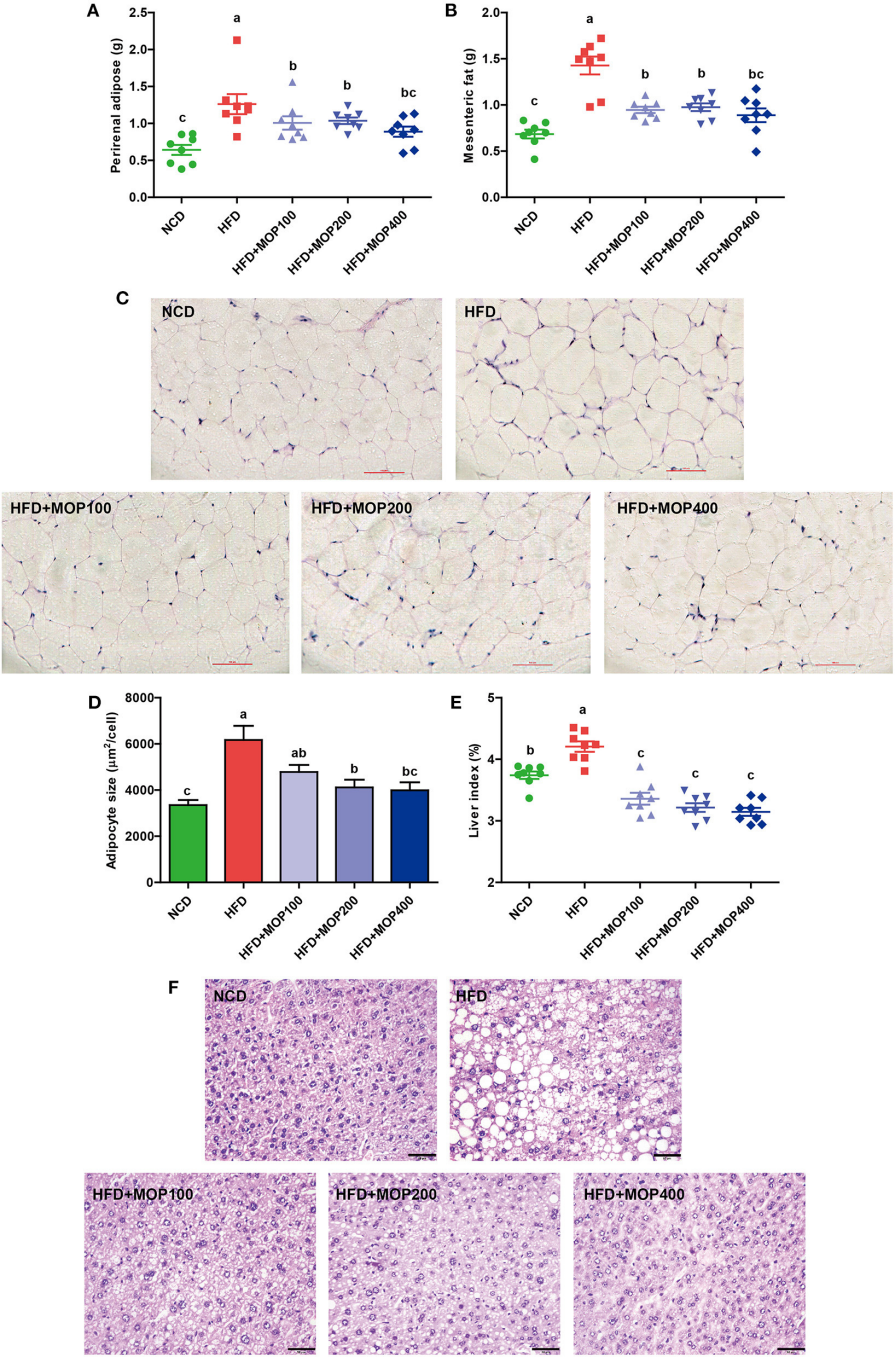
Preventive effects of MOP on fat accumulation in HFD-fed mice (*n* = 8 mice/group). **(A)** Perirenal fat weight, **(B)** Mesenteric fat weight, **(C)** Histological examination of perirenal fat by H & E staining, scale bar = 100 μm, **(D)** Adipocyte size was monitored in mesenteric fat tissue, **(E)** Liver index, **(F)** Representative liver histology by H & E staining, scale bar = 50 μm. Data are shown as means ± SEM. Bars marked with different superscript letters (a–c) indicate significant differences at *p* < 0.05 based on one-way ANOVA with Duncan's *post-hoc* test.

The effects of MOP on the adipose tissues were also examined after 12 weeks of treatment. Perirenal fat weight and mesenteric fat weight were induced by HFD and significantly reversed by MOP supplementation (Figures 2A,B). H & E staining showed an 84.12% increase in the adipocyte size of the HFD group. The average adipocyte size of the MOP-treated groups (especially at high-dose) was significantly reduced, which was similar to that of the normal diet group (Figures 2C,D), indicating that MOP could prevent HFD-induced adipose hypertrophy.

Fat accumulation in the liver is another important indicator of metabolic dysregulation induced by HFD. Therefore, the gross weight and histology of the liver from the different experimental groups were examined. Compared to the NCD group, the liver weights of HFD-fed mice were significantly increased. MOP supplementation remarkably reduced the liver index induced by HFD feeding, although there was no significant difference between the different MOP dose groups (Figure 2E). H & E staining of the liver revealed histological abnormalities of hepatocytes with larger fat vacuoles in the HFD group, indicating that the mice had suffered a higher degree of hepatic steatosis induced by HFD feding. MOP markedly reduced the size of the fat vacuoles in the liver induced by HFD (Figure 2F), suggesting that MOP exerted a liver protective effect by reducing fatty degeneration.

### MOP Ameliorates Blood Lipid Levels and Insulin Resistance

**Figure 3:**
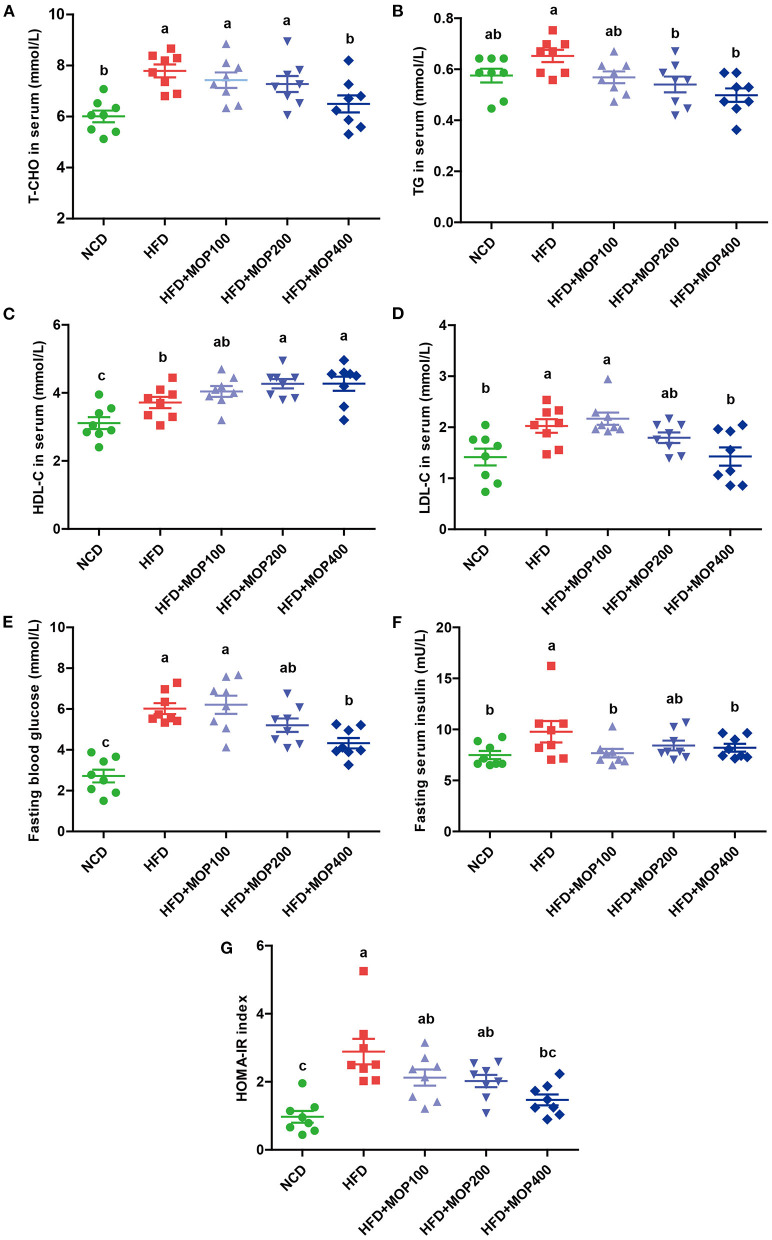
MOP ameliorates blood lipid levels and insulin resistance in HFD-fed mice (*n* = 8 mice/group). Serum levels of **(A)** total cholesterol (T-CHO), **(B)** triglycerides (TG), **(C)** high-density lipoprotein cholesterol (HDL-C), **(D)** low-density lipoprotein cholesterol (LDL-C), **(E)** Fasting blood glucose, **(F)** fasting serum insulin, **(G)** homeostasis model assessment of insulin resistance (HOMA-IR) index. Data are shown as means ± SEM. Bars marked with different superscript letters (a-c) indicate significant differences at *p* < 0.05 based on one-way ANOVA with Duncan's *post-hoc* test.

As excessive fat accumulation in obese individuals leads to dyslipidemia, lipid concentrations in serum were measured in the present study. Levels of total cholesterol (T-CHO), triglycerides (TG), high-density lipoprotein cholesterol (HDL-C), and low-density lipoprotein cholesterol (LDL-C) in serum were significantly higher in the HFD group, compared with those of the NCD group. In addition, MOP supplementation in HFD-fed mice significantly decreased the levels of T-CHO, TG, and LDL-C in a dose-dependent manner (Figures 3A–C). Notably, MOP supplementation significantly increased HDL-C concentrations (Figure 3D). These data suggest the protective effect of MOP against HFD-induced hypercholesterolemia and hyperlipidemia.

Obesity is closely correlated with impaired insulin signaling and is a major cause of the development of insulin resistance (39). Fasting glucose and fasting serum insulin were measured, and HOMA-IR was calculated in the present study. Fasting glucose and insulin levels were significantly elevated in HFD-fed mice, along with an increase in HOMA-IR values, and they were significantly reversed by MOP supplementation (Figures 3E–G). Apparently, MOP supplementation significantly decreased the insulin resistance induced by HFD.

### MOP Alleviates HFD-Induced Secretion of Pro-inflammatory Cytokines

**Figure 4:**
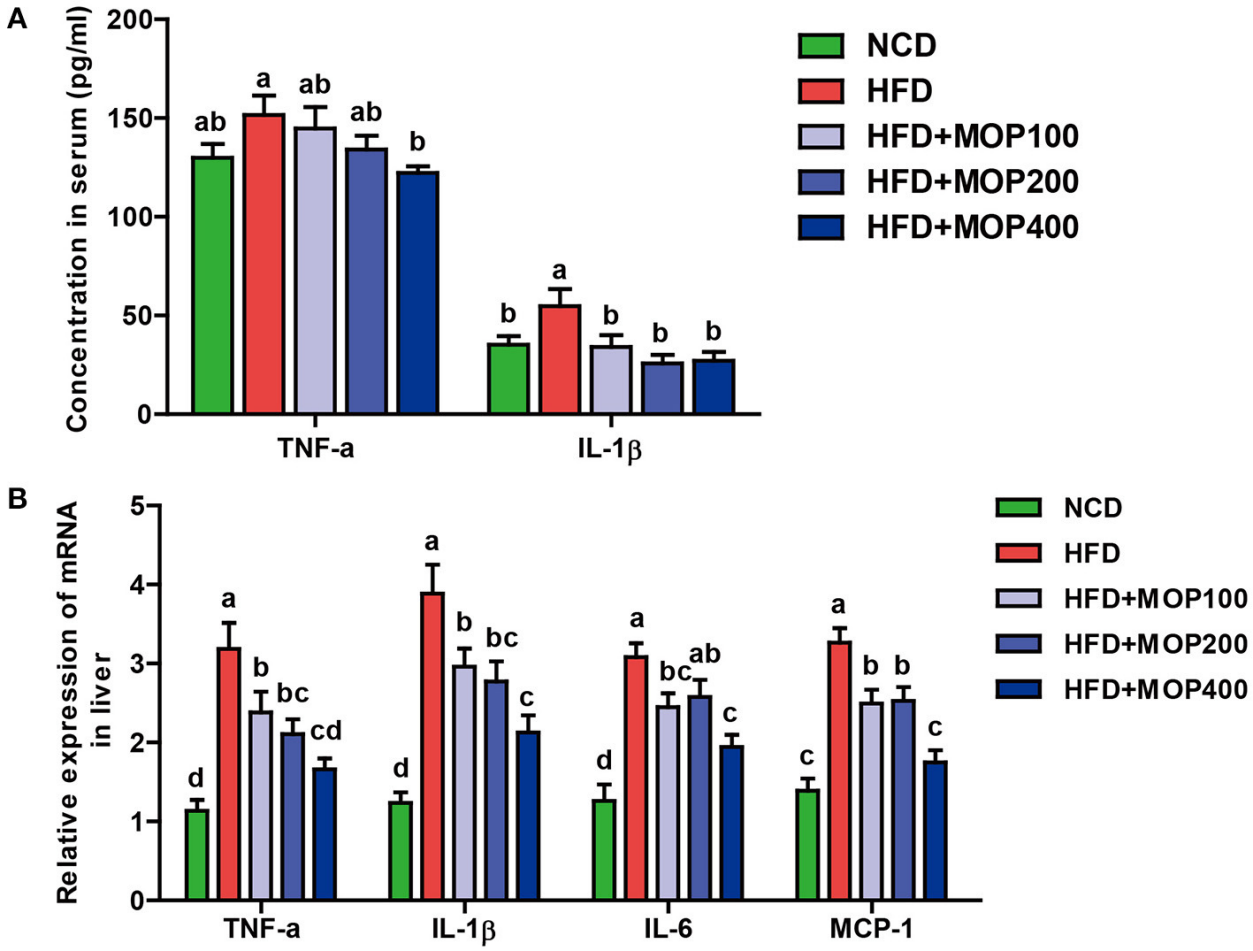
MOP alleviates the production of pro-inflammatory cytokines in HFD-fed mice. **(A)** Serum protein levels of TNF-α and IL-1β were determined by ELISA (*n* = 8 mice/group); **(B)** Relative mRNA expression levels of TNF-α, IL-1β, IL-6, and monocyte chemotactic protein-1 (MCP-1) in the liver as determined by qRT-PCR (*n* = 6). Bars marked with different superscript letters (a-d) indicate significant differences at *p* < 0.05 based on one-way ANOVA with Duncan's *post-hoc* test.

Chronic low-grade inflammation is one of the most important characteristics of obesity (40). To evaluate the anti-inflammatory effects of MOP, levels of representative pro-inflammatory cytokines including TNF-α and IL-1β were examined in mouse serum. Compared to the HFD group alone, MOP supplementation significantly restored serum concentrations of TNF-α and IL-1β back to normal (Figure 4A). Moreover, HFD significantly increased the mRNA expression of TNF-α, IL-1β, IL-6, and MCP-1 in the liver (Figure 4B). The expression of these pro-inflammatory cytokines was significantly reduced by MOP supplementation in a dose-dependent manner (Figure 4B). These results indicate that MOP supplementation can reduce HFD-induced systemic and liver inflammation.

### MOP Regulates the mRNA Expression Levels of Genes Involved in Lipid Metabolism and Bile Acid Synthesis in the Liver

**Figure 5:**
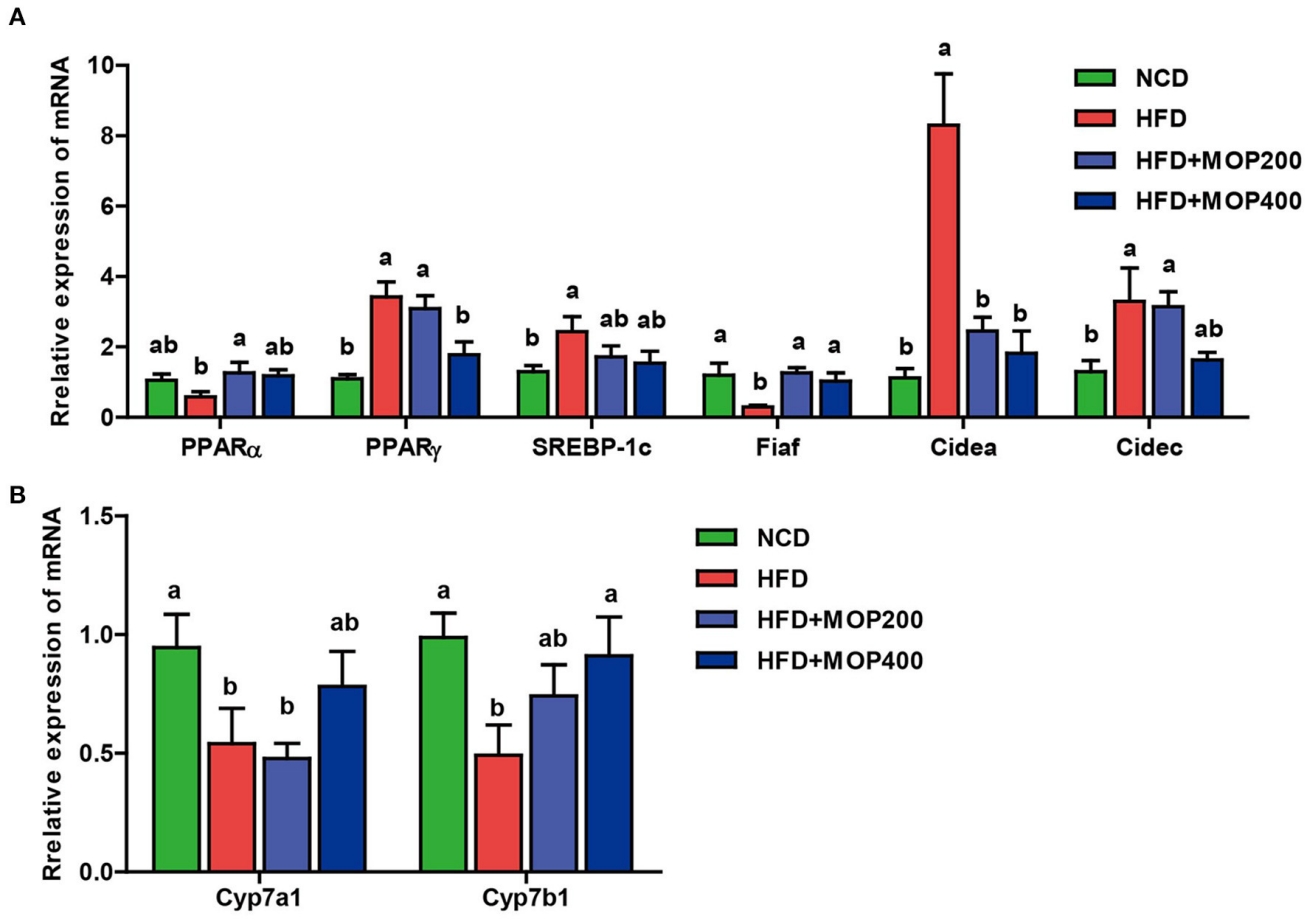
MOP regulates the mRNA expression levels of genes involved in lipid metabolism and bile acid synthesis in the liver. **(A)** Relative mRNA expression levels of genes involved in lipid synthesis; **(B)** Relative mRNA expression levels of genes involved in bile acids metabolism. The data are expressed as means ± SEM (*n* = 6). Bars marked with different superscript letters (a-c) indicate significant differences at *p* < 0.05 based on one-way ANOVA with Duncan's *post-hoc* test.

Previous studies have shown that synthesis and oxidation of the hepatic fatty acids might be regulated by gene expression after HFD treatment (41). Therefore, the effects of MOP on the expression levels of six genes related to fat metabolism in the liver, including PPARα, PPARγ, SREBP-1c, Fiaf, Cidea, and Cidec, were investigated. The results showed significantly higher expression of PPARγ, SREBP-1c, Cidea, and Cidec genes and lower expression of PPARα and Fiaf genes in the HFD group. The MOP intervention reversed the expression of these genes (Figure 5A). Moreover, bile acids play an important role in lipid metabolism and obesity. Also, Cyp7a1 and Cyp7b1 genes associated with bile acid metabolism were examined in this study. The expression levels of Cyp7a1 and Cyp7b1 genes were significantly reduced in HFD-fed mice, and the MOP intervention significantly increased the expression of Cyp7a1 and Cyp7b1 genes and was similar to the level of the NCD group (Figure 5B). These results suggest that MOP supplementation regulates the mRNA expression levels of genes involved in lipid metabolism and bile acid synthesis-related genes in the liver.

### MOP Reshapes the HFD-Induced Change in Gut Microbiota Profile

**Figure 6:**
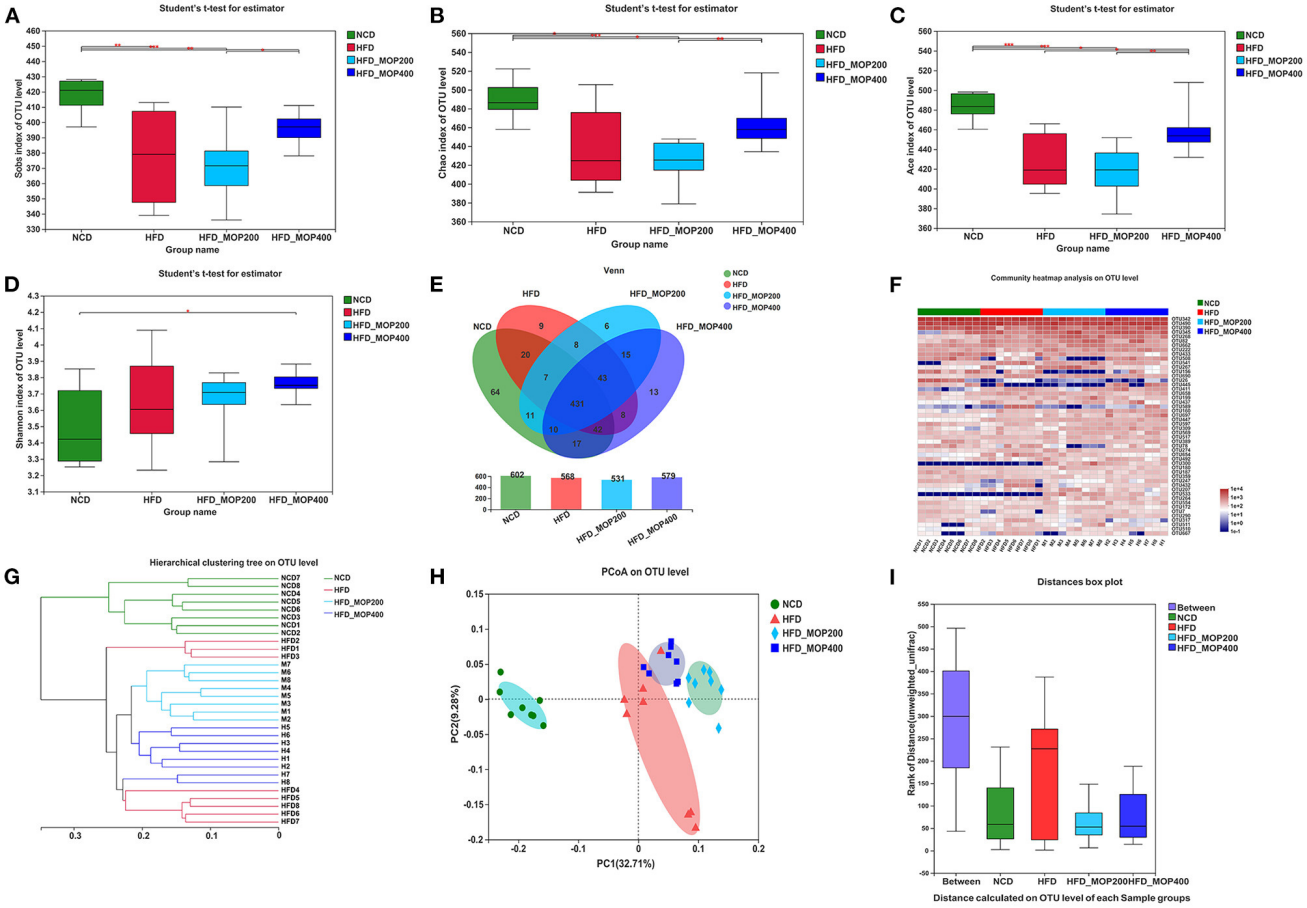
MOP alters the diversity and composition of the gut microbiota in HFD-fed mice (*n* = 8 mice/group). **(A–D)** Sobs index, Chao index, ACE index, and Shannon index, respectively; **(E)** Venn diagrams that illustrated the observed overlap of OTUs; **(F)** Heatmap of top 50 OTUs; **(G)** Hierarchical clustering dendrograms of all samples based on unweighted UniFrac distance; **(H)** PCoA plot of unweighted UniFrac distance; **(I)** Analysis of similarity (ANOSIM) using bray-curtis distance at OTU level. The data are expressed as mean ± SD. Bars marked with different superscript letters (a-c) indicate significant differences at *p* < 0.05 based on one-way ANOVA with Duncan's *post-hoc* test.

To evaluate the effect of MOP on the gut microbiota of HFD mice, the V3–V4 regions of the 16S rRNA gene were sequenced. A total of 1,830,065 raw sequences were obtained from 32 caecal content samples, each with more than 33,820 sequences for further analysis. These reads were clustered into 704 OTUs at a 97% sequence similarity level. The α-diversity analysis reflected the richness and diversity of the microbial community. In the present study, the community richness indices, including Sobs, Chao, and Ace indices, were all significantly lower in the HFD group than in the NCD group. To a certain extent, the high concentration of MOP (400 mg/kg/d) reversed the HFD-induced changes in these indices (Figures 6A–C). In addition, the Shannon index was higher in the two MOP-supplement groups than in the NCD group, indicating that MOP could increase the species diversity of the gut microbial profile (Figure 6D). The shared and specific OTUs among different groups are represented by Venn diagrams in Figure 6E. A total of 431 of 704 OTUs were shared among the four groups, with the NCD group having the largest number of unique OTUs (sixty-four). This was consistent with the results of the Sobs, Chao, and Ace indices, which showed that the NCD group had the highest values. An overview of the heatmap (Figure 6F) suggested a significant impact of MOP on gut microbiota profile. Furthermore, β-diversity parameters were analyzed to measure the distance between each sample and the similarities between the four experimental groups. Both the hierarchical clustering tree and principal coordinate analysis (PCoA) based on the unweighted UniFrac distance revealed that all four groups presented distinctive microbiota profiles, and a more similar structure was observed for the HFD-fed groups (Figures 6G,H). The analysis of similarity (ANOSIM) test using unweighted UniFrac distance showed that the observed clustering patterns were significant (*R* = 0.7697, *p* = 0.001) (Figure 6I).

**Figure 7:**
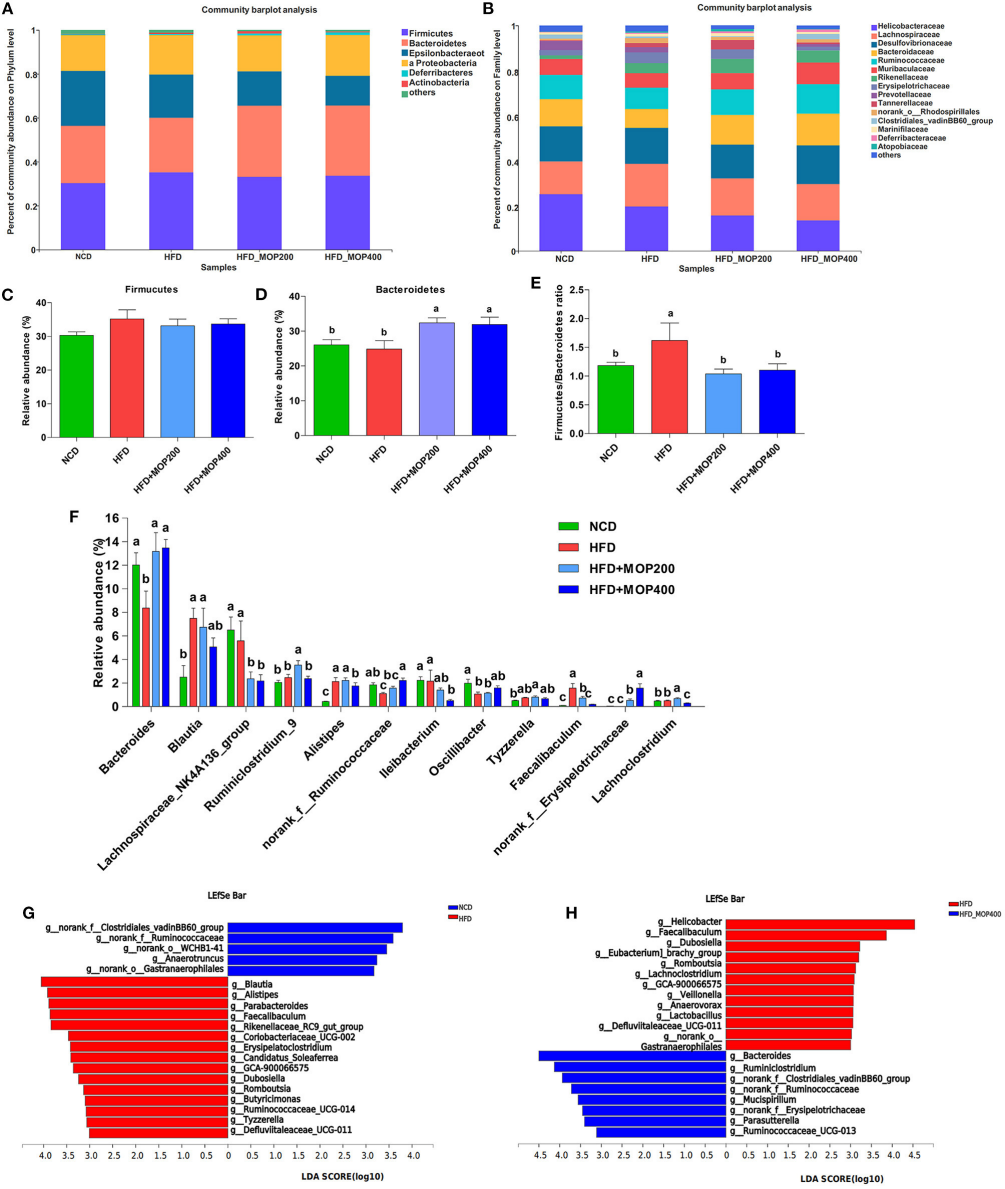
MOP affects the relative abundance of gut microbiota at the phylum and family level in HFD-fed mice (*n* = 8 mice/group). Bacterial taxonomic profiling at the phylum **(A)** and family **(B)** levels from different experimental groups. The relative abundance of Firmicutes **(C)** and Bacteroidetes **(D)**; **(E)** The Firmicutes to Bacteroidetes (F/B) ratio in different groups; **(F)** Comparisons of relative abundance of the bacterial genera. The LEfSe analysis of the gut microbiota differed between NCD and HFD groups **(G)**, and between HFD and HFD+MOP 400 groups **(H)**. The histogram showed the lineages with LDA values of 3.0 or higher as determined by LEfSe.

A closer look at the microbial community revealed a considerable positive influence of MOP at both the phylum and family levels (Figures 7A,B). At the phylum level, MOP showed a tendency to reduce the abundance of the Firmicutes, but a statistical significance difference was not achieved (Figure 7C). MOP treatment, however, significantly increased the abundance of Bacteroidetes (Figure 7D). Previous studies have shown an increase in the Firmicutes/Bacteroidetes (F/B) ratio in obese patients and in HFD-induced obese mice (42). Consistent with these studies, we also observed an increase in the F/B ratio in the HFD alone group compared to the NCD control group. It is noteworthy that MOP treatment apparently resorted the F/B ratio back to the NCD control group (Figure 7E), indicating a modulating effect on the gut microbiota. At the genus level, 12 of the top 30 genera in terms of abundance varied significantly between the four groups (Figure 7F). In particular, HFD significantly decreased the relative abundance of *Bacteroides*, norank_f_Ruminococcaceae, and *Oscillibacter*, while significantly increasing the relative abundance of *Blautia, Alistipes, Tyzzerella*, and *Faecalibaculum*. However, the intervention of MOP reversed the abundance of these bacteria.

To identify the key phylotypes that were significantly altered in response to MOP supplementation, all sequences of the four experimental groups were analyzed using the linear discriminant analysis effect size (LEfSe) method. At a threshold of 3.0 on the logarithmic LDA score, the LEfSe analysis revealed that highly enriched gut microbial taxa differed significantly between the 4 groups, with a total of 73 taxa differing significantly in abundance (see Supplementary Figure 1). Further LEfSe analysis revealed that HFD significantly affected 20 bacterial genera, of which the abundance of 5 genera was decreased, and the abundance of 15 genera was increased as compared to the NCD group (Figure 7G). The top 5 genera enriched by HFD feeding were *Blautia, Alistipes, Parabacteroides, Faecalibaculum*, and Rikenellaceae_RC9_gut_group. A 12-week MOP intervention also significantly affected 20 bacterial genera, of which the abundance of 8 genera was increased, and the abundance of 12 genera was decreased (Figure 7H). Among these species, the enrichment of *Bacteroides* and *Ruminiclostridium*, and the inhibition of *Helicobacter, Faecalibaculum, Dubosiella*, and *Romboutsia* were notable.

### Potential Relations Between Gut Microbiota and Obesity-Related Biomarkers

**Figure 8:**
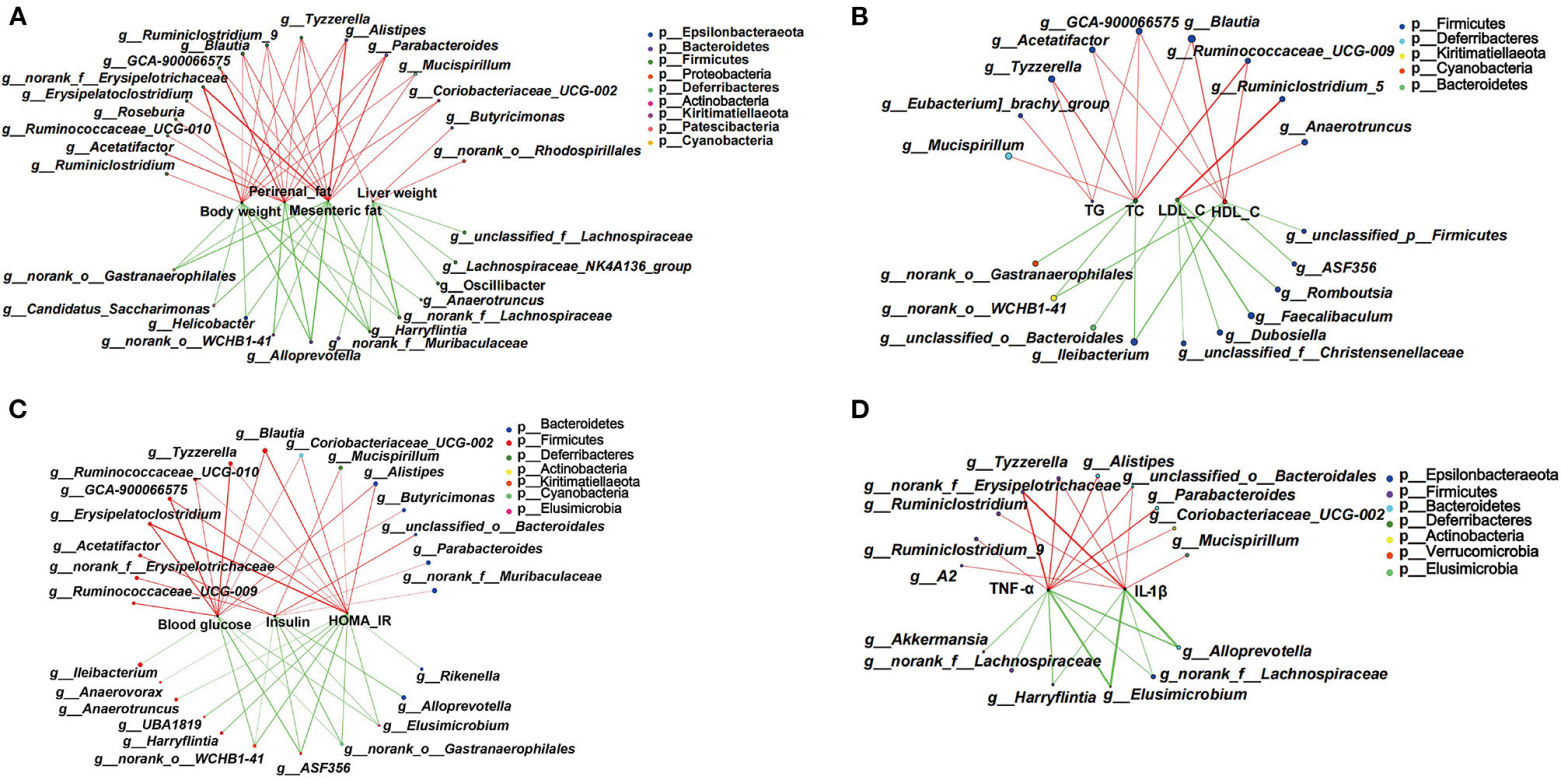
Two-factor correlation network analysis showing correlations between specific gut microbiota and obesity-related biomarkers (*n* = 8 mice/group). **(A)** Correlations between gut bacteria and weight parameters, including body weight, mesenteric fat, perirenal fat, and liver weight. **(B)** Correlation between gut bacteria and blood lipid profile of TG, T-CHO, LDL-C, and HDL-C in serum. **(C)** Correlations between gut bacteria and glucose hemostasis, including fasting blood glucose, insulin, and the HOMA-IR index. **(D)** Correlations between gut bacteria and pro-inflammatory cytokines including TNF-α and IL-1β in serum. Spearman's r coefficient, |r| of ≥ 0.5, *p* < 0.05. Red lines represent r ≥ 0.5, and green lines represent r ≤ −0.5.

Based on the significant improvement of obesity-related symptoms and gut microbiota in HFD mice by MOP, the two-factor correlation network analysis was used to establish the relations between gut microbiota and obesity-related parameters (Figures 8A–D). As shown in Figure 8A, the correlations between 28 specific gut bacterial genera and 4 weight parameters (body weight, mesenteric fat, perirenal fat, and liver weight) were clearly visible. *Tyzzerella* was the only genus that was significantly positively correlated with all four weight parameters. *Blautia, Ruminiclostridium*_9, *Alistipes*, and *Parabacteroides* were positively correlated with three of the weight parameters. However, *Harryflintia* was significantly negatively correlated with all four weight parameters, while *Alloprevotella*, norank_o_Gastranaerophilales, and norank_f__Lachnospiraceae were negatively correlated with three of the weight parameters.

The correlations between specific gut bacterial genera and lipid-related parameters are shown in Figure 8B. The unclassified GCA-900066575 was the only genus with significant positive correlations for TG, T-CHO, and HDL-C. *Tyzzerella, Acetatifactor, Blautia*, and Ruminococcaceae_UCG-009 were significantly positively correlated with two of the lipid parameters. However, *Ileibacterium* and norank_o_WCHB1-41 were significantly negatively correlated with T-CHO and HDL-C. Notably, those taxa significantly correlated with LDL-C were not correlated with TG, T-CHO, and HDL-C.

Some specific genera of gut bacteria were closely associated with glucose homeostasis (Figure 8C). Six genera such as *Erysipelatoclostridium, Tyzzerella*, and *Blautia* were significantly positively associated with blood glucose and HOMA-IR. *Mucispirillum* and *Alistipes* were significantly negatively associated with insulin and HOMA-IR. Notably, *Elusimicrobium*, norank_o_Gastranaerophilales, g_ASF356, and norank_o__WCHB1-41 showed significant negative correlations with all three glucose homeostatic parameters.

The correlations between specific gut bacteria and pro-inflammatory cytokines are shown in Figure 8D. *Tyzzerella, Alistipes*, norank_f_Erysipelotrichaceae, and unclassified_o_Bacteroidales were significantly positively correlated with TNF-α and IL-1β. In contrast, *Harryflintia, Elusimicrobium, Alloprevotella*, and norank_f_Lachnospiraceae were significantly negatively correlated with these two pro-inflammatory cytokines. These correlations suggested that gut microbiota could influence not only host phenotypes, but also serum lipid-related parameters, glucose metabolism, and inflammatory markers.

## Discussion

The prevalence of overweight and obesity is an increasing chronic disease worldwide, and has become a growing global health problem. In recent years, some studies have shown that extracts from *M. oliefera* leaves could regulate lipid metabolism. For example, Syamsunarno et al. (43) explored the effects of ethanol extract from *M. oleifera* leaves on modulating brown adipose tissue differentiation in mice fed an HFD. Mabrouki et al. (44) evaluated the effect of the methanolic extract of *M. oleifera* leaves on HFD-induced obesity and cardiac damage in rats. Sha et al. (45) investigated the efficacy and mechanism of new resource medicinal material *M. oleifera* leaves against hyperlipidemia. Our team has also investigated *M. oleifera* leaf petroleum ether extract inhibits lipogenesis (46). In these studies, the main components of *M. oleifera* leaf extracts were phenolic compounds (44) and flavonoid glycosides (46), without even elucidating their main components (43, 45). Therefore, the activity of MOP in regulating lipid metabolism is currently unclear. Furthermore, there has been growing evidence that the occurrence of obesity is closely related to alteration in the gut microbiota. Thus, growing interests have been aimed to modulate gut microbiota as a therapeutic strategy against obesity and its related diseases (11, 12). Many studies have shown that bioactive components of plants, including kale (47), *Ganoderma lucidum* (42), *Lobelia chinensis* (48), and *Dictyophora indusiata* (3), can inhibit the development of obesity in experimental animals by regulating gut microbiota. However, the biological activities of MOP in modulating gut microbiota is also unclear. The present study demonstrated that crude polysaccharides extracted from *M. oleifera* leaves could effectively prevent weight gain, fat accumulation, lipid increase, and chronic inflammation in HFD-induced obese mice, which were associated with modulation of the gut microbiota. This is the first report to assess the activity of MOP for obesity prevention, and to investigate the regulation of the gut microbial community by MOP. These findings suggested that easily available MOP could be served as an alternative strategy for preventing obesity and obesity-related metabolic diseases.

Our study found that MOP supplementation significantly reduced fat weight and adipocyte size in HFD-induced obese mice (Figures 2A–D). Meanwhile, the size of fat vacuoles in the liver gradually decreased with the increase of MOP dose, suggesting that MOP helped reduce fat accumulation to protect the liver (Figure 2F). Previous studies have shown that the feeling of satiety is increased due to the swelling effect of natural polysaccharides, and thus reduced feeding occurs during the ingestion of undigested polysaccharides (49). Our results showed that MOP supplementation did not reduce daily food intake or caloric intake in mice (Figures 1C,D), suggesting that the protective effect of MOP on HFD-induced weight gain was not achieved by suppressing food or energy intake. Abnormal lipid metabolism is one of the most common characteristics of obesity and related chronic diseases. Our study found that MOP significantly reduced serum TG, T-CHO, and LDL-C concentrations, and increased HDL-C concentrations in a dose-dependent manner, compared to HFD-fed mice (Figures 3A–D). This is consistent with the results of previous studies (50, 51). These results suggest that MOP probably plays an influential role in reducing obesity-induced abnormal lipid metabolism and dyslipidemia.

Obesity is a major cause of impaired insulin signaling and, therefore, insulin resistance development (52). Insulin resistance leads to impaired glycogen synthesis and proteolytic metabolism in skeletal muscle, and inhibits lipoprotein lipase activity in adipocytes, resulting in the release of free fatty acids and inflammatory cytokines. In addition, insulin resistance could lead to impaired glucose output and fatty acid metabolism, resulting in increased triglyceride levels and hepatic VLDL secretion (53). Hyperglycemia is a hallmark of insulin resistance (54). In this study, MOP supplementation reduced fasting blood glucose and fasting serum insulin in HFD-fed mice (Figures 3E–G), indicating a positive effect of MOP on improving insulin resistance.

Previous studies have shown that obesity is associated with inflammation. Obesity is regarded as a chronic low-grade inflammatory state (55). To assess the anti-inflammatory effects of MOP, the levels of representative pro-inflammatory cytokines including TNF-α and IL-1β in serum, and the mRNA expression of TNF-α, IL-1β, IL-6, and MCP-1 in the liver were examined. The results showed that these pro-inflammatory cytokines were significantly reduced by MOP intervention in a dose-dependent manner (Figure 4), indicating that MOP supplementation could reduce HFD-induced systemic inflammation and liver inflammation. This is similar to the findings of Sang et al. (42), who found that *Ganoderma lucidum* polysaccharides significantly inhibited HFD-induced inflammatory response, manifested in the reduction of inflammatory factors (TNF-α, IL-1β, and MCP-1) in serum and adipose tissue, and reduced macrophage infiltration in adipose tissue.

As previously reported, HFD can promote obesity by altering the expression of lipid-related genes in the liver (56). Peroxisome proliferator-activated receptors (PPARs) are ligand-activated transcription factors that belong to the nuclear receptor superfamily (57). PPARs have been considered potential therapeutic targets for treating several metabolic syndromes (58). PPARs, including PPARα, PPARγ, and other subtypes, could regulate the expression of many genes, involved in lipid metabolism, adipogenesis, inflammation, apoptosis, immune response, oxidative stress, and adipocyte differentiation (59). PPARα is highly expressed in tissues associated with increased fatty acid oxidation (e.g., liver, skeletal muscle, and heart), and its activation leads to lower lipid levels and the elimination of TG in plasma, resulting in high levels of HDL-C (60). In contrast, PPARγ is a transcription factor involved in adipocyte differentiation and adipogenesis, which includes the processes of preadipocyte recruitment, differentiation, and TG synthesis (50). In the present study, we found by RT-qPCR analysis that MOP treatment resulted in increased expression of PPARα and decreased expression of PPARγ in the liver, compared to the HFD group (Figure 5A). SREBP-1c could regulate the expression of fatty acid synthase, participate in hepatic adipose differentiation and adipogenesis (61, 62). In this study, MOP supplementation decreased the expression of the SREBP-1c gene (Figure 5A). Fasting-induced adipose factor (Fiaf) is a multifunctional protein involved in plasma triglyceride (TG) metabolism, energy metabolism, cancer metastasis, angiogenesis, wound healing, inflammation, and nephrotic syndrome (63). Increased levels of Fiaf have been shown to be protective against diet-induced obesity (64). In the present study, MOP treatment significantly decreased the gene expression of Fiaf compared to HFD (Figure 5A). The Cide (cell death-inducing DFF45-like effector) family of proteins has been shown to play a critical role in lipid metabolism and energy metabolism, including lipolysis, lipid oxidation, and lipid droplet formation (65). The expression levels of Cidea and Cidec genes were examined in this study, and it was found that MOP treatment decreased their expression levels (Figure 5A). Bile acids are critical to lipid digestion, cholesterol metabolism, and other lipid-related pathways (66). Increased primary bile acid biosynthesis has been shown to be associated with obesity (67). Cyp7a1 is a rate-limiting enzyme for cholesterol catabolism and bile acid synthesis (68). Cyp7b1 is a microsomal enzyme involved in various physiological functions, including bile acid biosynthesis (69). Several studies have confirmed that an HFD significantly reduces the gene expression of Cyp7a1 and Cpy7b1 in the liver (70). In this study, MOP supplementation increased Cyp7a1 and Cyp7b1 expression in the liver compared to the HFD group, suggesting that MOP may prevent obesity by regulating bile acid metabolism (Figure 5B). This is consistent with the results of a previous study (71), in which a significant increase was found in the mRNA expression levels of Cpy7a1 and Cyp7b1 in the liver after theabrownin treatment. In conclusion, our findings suggested that MOP regulated the expression levels of genes related to hepatic fatty acid synthesis and lipid metabolism, thereby preventing obesity.

The gut microbiota depends heavily on plant-derived dietary fiber and polysaccharides as energy sources. Besides, dietary polysaccharides significantly impact the host's gut microbial ecology and health (42). Some studies have shown that the gut microbiota is an environmental factor associated with obesity (72, 73). There is growing evidence that dysbiosis of the gut microbiota is associated with the development of obesity. However, emerging evidence supports the benefits of gut microbiota in weight management (74). Many natural products, including plant foods and phytochemicals, have been found to be effective in weight management by modulating gut microbiota (75). Several studies have also found that the prevention of obesity by plant polysaccharides is related to the regulation of gut microbiota (3, 42, 76). The decrease in microbial diversity and abundance is one of the main features of human obesity-related gut malnutrition (77). The present study showed that HFD reduced the diversity indices of gut microbiota, including Sobs, Chao, and Ace, while high concentrations of MOP (400 mg/kg/d) reversed the HFD-induced changes in these indices to some extent. Moreover, MOP supplementation increased the species diversity of the gut microbiota (Figures 6A–D). It is probably explained that dietary polysaccharides, which are usually indigestible in the stomach and small intestine, reach the large intestine, where they are available for fermentation, thereby enhancing the growth of beneficial bacteria and improving gut microbial diversity.

The human gut microbiota is dominated by two phyla, Firmicutes and Bacteroidetes, which account for more than 90% of all bacterial species in the gut (78). These two bacteria contribute to the host's energy absorption and metabolism associated with gut microbiota. The F/B ratio was confirmed to be higher in obese individuals compared with the healthy population (56). Although many studies point toward a relative increase in the F/B ratio as a characteristic of the “obese microbiome,” the findings are inconsistent. For example, some studies reported that polyphenols or herbal extracts with anti-obesity bioactivity could downregulate the F/B ratio (9), whereas the protective effect of berberine against HFD-induced obesity was not associated with any significant change in the F/B ratio (79). In the current study, MOP supplementation did not significantly reduce the abundance of Firmicutes, but significantly increased the abundance of Bacteroidetes and the F/B ratio (Figures 7C–E). The results of this study showed that MOP treatment helped maintain the F/B ratio at a level similar to that of the NCD group, thereby helping to control energy absorption and body weight in the HFD mice.

Our results showed that MOP treatment altered the abundance of some bacterial genera (Figure 7F). For instance, the abundance of *Bacteroides* and *Oscillibacter* was increased. *Bacteroides* is a major genus in the human microbiota with a broad ability to use various types of dietary polysaccharides (42). Kaoutari et al. reported that members of the Bacteroidetes encode a proportionally higher number of carbohydrate-active enzymes (CAZymes) that are key enzymes for digesting polysaccharides than bacteria of other phyla (80). *Oscillibacter* is considered a potentially beneficial bacterium (81). Studies have shown that *Oscillibacter* is a valeric acid producer that enhances the differentiation of interleukin 10 (IL-10)-producing Tregs *in vivo* (82). Kim et al. (83) investigated the effect of *Ephedra sinica* on gut microbiota composition, and found that *Oscillibacter* in Firmicutes was negatively correlated with body weight and body mass index (BMI). These studies support our findings that MOP treatment increased the abundance of *Bacteriodes* and *Oscillibacter*. Furthermore, our results showed that MOP treatment reduced the relative abundance of *Blautia* compared to HFD (Figure 7F). *Blautia* is a commonly present genus in the gut microbiota. However, the results of studies on the relationship between *Blautia* and obesity are inconsistent. In a Japanese weight loss study, the genus *Blautia* was observed as the only gut microbe that was inversely associated with visceral fat, independent of sex (84). In contrast, some studies have suggested that *Blautia* is a harmful bacterium (85). In the current study, the results of LFSE analysis showed that *Blautia* was an important factor in distinguishing HFD and NCD groups (Figure 7G). Moreover, our findings showed that *Blautia* was positively associated with most obesity markers, including body weight, perirenal fat, mesenteric fat, T-CHO, HDL-C, blood glucose, and HOMA-IR (Figures 8A–C). Our findings are consistent with those of Goffredo et al., who reported a positive correlation between the abundance of *Blautia* and obesity in American youth and verified that the level of acetate, which is the product by *Blautia*, was associated with body fat partitioning and hepatic lipogenesis (86). *Alistipes* is considered to be an obesity-associated bacterium. Previous studies showed that HFD significantly increased the abundance of *Alistipes* in feces (87). In this study, MOP treatment reduced the abundance of *Alistipes* (Figure 7F). This is consistent with the previous study, in which the probiotic-fermented blueberry juice treated mice showed relatively low abundances of obese-related gut bacteria (*Oscillibacter* and *Alistipes*) (88). In a randomized controlled trial, overweight and obese adults consuming avocado daily for 12 weeks were enriched in the relative abundance of *Alistipes* compared to the control group (89). *Tyzzerella* is considered a pathogenic bacteria in the intestine (90). Huang et al. investigated the protective effects of sodium alginate on the gut microbiota, immunity, and intestinal mucosal barrier function in cyclophosphamide-induced immunosuppressed BALB/c mice, and found that sodium alginate significantly decreased the pathogenic bacteria *Tyzzerella* in the intestine (90). However, few studies have reported the correlation between *Tyzzerella* and obesity. The current study showed that MOP treatment reduced the relative abundance of *Tyzzerella* (Figure 7F). Furthermore, *Tyzzerella* was positively correlated with the majority of the obesity indicators, including body weight, liver weight, perirenal fat, mesenteric fat, TG, T-CHO, blood glucose, HOMA-IR, TNF-α, and IL-1β (Figure 8). These results suggested that *Tyzzerella* is a “harmful indicator” closely related to obesity.

In conclusion, this study demonstrated that MOP could effectively prevent HFD-induced weight gain and lipid accumulation, ameliorate blood lipid levels and insulin resistance, and reduce the secretion of inflammatory cytokines in mice. In addition, MOP supplementation also modulated gut microbiota composition, increasing the abundance of some beneficial bacteria and decreasing the abundance of some harmful bacteria. These results might be attributed to the change in gut microbial composition and regulation of lipid metabolism induced by the prebiotic ability of polysaccharides. This study suggests that MOP possesses great potential to be utilized as a dietary supplement for obesity management. In future, it would be important to confirm the composition of polysaccharides in *M. oleifera* leaves and to identify specific ones with anti-obesity properties.

## Data Availability Statement

The original contributions presented in the study are publicly available. This data can be found here: NCBI, PRJNA759102.

## Ethics Statement

The animal study was reviewed and approved by the Animal Care and Use Committee of Yunnan Agricultural University.

## Author Contributions

JS, YT, and LM conceived and designed the experiments. LL, LM, YW, JX, LY, AJ, and YZ performed the experiments. YT, LL, and LM analyzed the data. LL and LM wrote the paper. All authors contributed to the article and approved the submitted version.

## Funding

This work was supported by Major Project of Science and Technology Department of Yunnan Province (2018ZI001 and 202002AA100005), YEFICRC Project of Yunnan Provincial Key Programs (2019ZG009), and Yunnan Province Young and Middle-Aged Academic and Technical Leaders Reserve Talents Project (2018HB040).

## Conflict of Interest

The authors declare that the research was conducted in the absence of any commercial or financial relationships that could be construed as a potential conflict of interest.

## Publisher's Note

All claims expressed in this article are solely those of the authors and do not necessarily represent those of their affiliated organizations, or those of the publisher, the editors and the reviewers. Any product that may be evaluated in this article, or claim that may be made by its manufacturer, is not guaranteed or endorsed by the publisher.
