# Supplementary material for: Crude Polysaccharide Extracted From Moringa oleifera Leaves Prevents Obesity in Association With Modulating Gut Microbiota in High-Fat Diet-Fed Mice
Source: Front Nutr. 2022 Apr 25;9:861588. doi: 10.3389/fnut.2022.861588 (PMC9083904; doi:10.3389/fnut.2022.861588)
Supplement: Supplementary file 1 [file Table_1.DOCX]

Supplementary Material

Crude polysaccharide extracted from Moringa oleifera leaves prevents obesity in association with modulating gut microbiota in high-fat diet-fed mice

**Supplementary Table 1:** Primer information of detected genes

| **Primers** | **Forward sequence** | **Reverse sequence** |
| --- | --- | --- |
| PPARα | GAGCTGCAAGATTCAGAAGAAG | GAATCTTTCAGGTCGTGTTCAC |
| PPARγ | CCAAGAATACCAAAGTGCGATC | TCACAAGCATGAACTCCATAGT |
| SREBP-1c | GATGTGCGAACTGGACACAG | CATAGGGGGCGTCAAACAG |
| Fiaf | AAAAGATGCACCCTTCAAAGAC | CTTGAGCTGAGTCTGCAAACTC |
| Cidea | CAATGTCAAAGCCACGATGTAC | CTGTGCAGCATAGGACATAAAC |
| Cidec | CAAGGTCCAGGACATCTTGAAA | ATTCTTCTGTCTCCACGATTGT |
| Cyp7a1 | GTGATGTTTGAAGCCGGATATC | TTTATGTGCGGTCTTGAACAAG |
| Cyp7b1 | AACCCTTTCCAGTACCAGTATG | GTGAACGTCTTCATTAAGGTCG |
| TNF-α | AGACCCTCACACTCAGATCA | TCTTTGAGATCCATGCCGTTG |
| IL-1β | TCCATGAGCTTTGTACAAGGA | AGCCCATACTTTAGGAAGACA |
| IL-6 | GTTCTCTGGGAAATCGTGGA | TGTACTCCAGGTAGCTA |
| MCP-1 | TTAAAAACCTGGATCGGAACCAA | GCATTAGCTTCAGATTTACGGGT |
| β-actin | CTACCTCATGAAGATCCTGACC | CACAGCTTCTCTTTGATGTCAC |
