# Supplementary figures and images for: Crude Polysaccharide Extracted From Moringa oleifera Leaves Prevents Obesity in Association With Modulating Gut Microbiota in High-Fat Diet-Fed Mice
Source: Front Nutr. 2022 Apr 25;9:861588. doi: 10.3389/fnut.2022.861588 (PMC9083904; doi:10.3389/fnut.2022.861588)

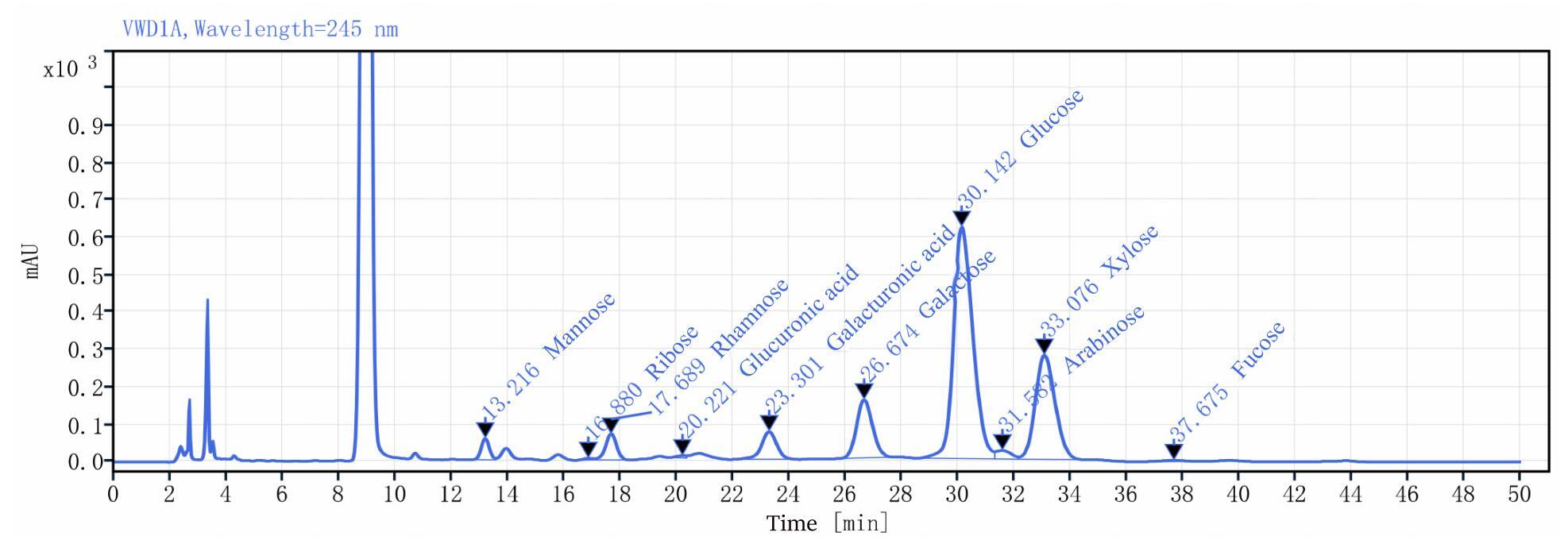

Supplement: Supplementary file 2 [file Image_1.JPEG]
